# 502. Support Engagement After Burn Injury: High Impact, High Desire, Low Participation

**DOI:** 10.1093/jbcr/irag033.161

**Published:** 2026-04-06

**Authors:** Sara E Byrne, Daniel W Chacon, Amy L Acton, Jill L Sproul, Sandra J Cramolini, Rachel Kudlak, Liliana Palacios

**Affiliations:** Phoenix Society for Burn Survivors, Grand Rapids, MI; Phoenix Society for Burn Survivors, Fresno, CA; Phoenix Society for Burn Survivors, Kentwood, MI; Phoenix Society for Burn Survivors, San Jose, CA; Phoenix Society for Burn Survivors, Knoxville, TN; Phoenix Society for Burn Survivors, Oakland, CA

## Abstract

**Introduction:**

Burn support programming is central to recovery, offering emotional, informational, and social benefits. Survivors report strong desire for connection and advocacy, yet participation remains limited. This study examines the disconnect between desire, participation, and outcomes in long-term recovery.

**Methods:**

A cross-sectional, self-report online survey was completed by burn survivors. Items analyzed included: support engagement (receiving/offering peer support), support group attendance (in-person/virtual), survivor event participation, perceived impact and benefits, expressed desires and needs, recovery phase, time since injury, age at injury, visible scarring, and demographics (language, education, race/ethnicity, income). Data were descriptively analyzed; n values are reported per item due to varying response rates. Demographics, burn characteristics, and recovery phase were reviewed as potential modifiers.

**Results:**

Participation. In 2024, only 29% (n = 102) received peer support and 21% (n = 81) offered support. Support group participation was low: 26% (n = 98) in-person and 30% (n = 113) virtual; percentages reflect any past-year attendance (≥1 session), and 68% (n = 260) reported no group attendance. Only 24% (n ≈ 83) joined a survivor gathering/camp.

Impact. Among those receiving support, 93% (n = 95) reported positive influence and 100% (n = 102) would recommend. Reported benefits included less isolation (84%, n = 80), more hope (82%, n = 78), and new coping skills (71%, n = 67).

Desire and needs. Interest was high: 62% (n = 262) wanted to give back and 65% (n = 273) to share their story, yet only 21% (n = 81) had served as supporters. Top needs included coping tools for chronic conditions (95%, n = 344) and survivor connection (95%, n = 343).

Context. Most respondents were in Recovery Phase 4 (“connected to my resilience and living my life”) (70%, n = 291), ≥10 years post-injury (53%, n = 216), and nearly all had visible scarring (99%, n = 407). Over half were injured before age 20 (52%, n = 210).

Other variables. Demographic and injury-related factors showed no significant associations with participation or perceived outcomes.

**Conclusions:**

Despite strong endorsement and clear benefits, participation across support modalities remains low. Survivors express high desire to connect, give back, and advocate, yet relatively few engage in one-to-one support, groups, or events. Converting interest into participation will likely require increased awareness, reduced access barriers, and more flexible delivery models; future work should identify modifiable barriers in long-term recovery.

**Applicability of Research to Practice:**

Programs should expand outreach, reduce logistical and perceptual barriers, and diversify delivery. Survivor-identified priorities can guide development to increase engagement and reach those most likely to benefit; additional research should clarify barriers such as accessibility and misconceptions.

**Funding for the study:**

N/A.

